# Drug-Repurposing Strategy for Dimethyl Fumarate

**DOI:** 10.3390/ph16070974

**Published:** 2023-07-07

**Authors:** Salvatore Giunta, Agata Grazia D’Amico, Grazia Maugeri, Claudio Bucolo, Giovanni Luca Romano, Settimio Rossi, Chiara M. Eandi, Elisabetta Pricoco, Velia D’Agata

**Affiliations:** 1 Department of Biomedical and Biotechnological Sciences, University of Catania, 95123 Catania, Italy; sgiunta@unict.it (S.G.); graziamaugeri@unict.it (G.M.); giovanniluca.romano@unict.it (G.L.R.); vdagata@unict.it (V.D.); 2 Department of Drug and Health Sciences, University of Catania, 95123 Catania, Italy; agata.damico@unict.it; 3 Center for Research in Ocular Pharmacology (CERFO), University of Catania, 95123 Catania, Italy; 4 Eye Clinic, Multidisciplinary Department of Medical, Surgical and Dental Sciences, University of Campania “Luigi Vanvitelli”, 80131 Napoli, Italy; settimio.rossi@unicampania.it; 5 Department of Ophthalmology, University of Lausanne, Fondation Asile des Aveugles, Jules Gonin Eye Hospital, 1004 Lausanne, Switzerland; chiara.eandi@unito.it; 6 Department of Medical and Surgical Sciences and Advanced Technologies “G. F. Ingrassia”, University of Catania, 95123 Catania, Italy; elisabettap@tiscali.it

**Keywords:** dimethyl fumarate, diabetic retinopathy, streptozotocin, retina, heme oxygenase-1

## Abstract

In the area of drug discovery, repurposing strategies represent an approach to discover new uses of approved drugs besides their original indications. We used this approach to investigate the effects of dimethyl fumarate (DMF), a drug approved for relapsing–remitting multiple sclerosis and psoriasis treatment, on early injury associated with diabetic retinopathy (DR). We used an in vivo streptozotocin (STZ)-induced diabetic rat model. Diabetes was induced by a single injection of STZ in rats, and after 1 week, a group of animals was treated with a daily intraperitoneal injection of DMF or a vehicle. Three weeks after diabetes induction, the retinal expression levels of key enzymes involved in DR were evaluated. In particular, the biomarkers COX-2, iNOS, and HO-1 were assessed via Western blot and immunohistochemistry analysis. Diabetic rats showed a significant retinal upregulation of COX-2 and iNOS compared to the retina of normal rats (non-diabetic), and an increase in HO-1 was also observed in the STZ group. This latter result was due to a mechanism of protection elicited by the pathological condition. DMF treatment significantly induced the retinal expression of HO-1 in STZ-induced diabetic animals with a reduction in iNOS and COX-2 retinal levels. Taken together, these results suggested that DMF might be useful to counteract the inflammatory process and the oxidative response in DR. In conclusion, we believe that DMF represents a potential candidate to treat diabetic retinopathy and warrants further in vivo and clinical evaluation.

## 1. Introduction

Diabetic retinopathy (DR) is a complication of diabetes characterized by vascular and inflammatory issues, sometimes associated with an alteration in the ocular surface [1,2]. Ocular inflammation and oxidative stress play important roles in the pathogenesis of DR, leading to changes in retinal microcirculation and blood–retinal barriers, as shown in animal models and in patients [1]. Given the inflammatory role of early diabetic retinopathy, we believe that drugs such as dimethyl fumarate (DMF) would constitute an attractive candidate for DR. DMF is a methyl ester of fumaric acid (FA), belonging to the family of fumaric acid esters, molecules identified in nature in the *Fumaria officinalis* plant. This molecule has a molecular weight of 144, and it is an α, β-unsaturated carboxylic ester [3]. DMF has anti-inflammatory and immunomodulatory properties and has been approved for relapsing–remitting multiple sclerosis and psoriasis treatment.

DMF is a well-tolerated molecule with well-characterized mechanisms of action [3]. DMF also has strong potential in eye diseases and might be used in several ocular conditions [4]. The primary pharmacodynamic response to DMF treatment is the activation of the heme oxygenase-1 (HO-1) gene via the regulation of nuclear factor erythroid-2-related factor-2 (Nrf2). Much evidence has indicated the critical importance of upregulating HO-1 in mediating anti-inflammatory and antioxidant effects. Therefore, HO-1 overexpression via pharmacologic modulation or gene transfer may represent a novel strategy for a therapeutic approach in the future. In the eye, hemin administration and its following over-expression protect the retina in streptozotocin-induced diabetic rats, reduce inflammation, and promote corneal wound healing [5]. From a therapeutic perspective, several molecules possess the ability to activate the Nrf2/HO-1 axis and reduce inflammation in the retina [6,7], suggesting that the identification of interesting compounds that enhance the expression of endogenous antioxidant proteins in retinal tissue may have therapeutic value in the treatment of ocular diseases. In our previous in vitro studies, we demonstrated that the upregulation of HO-1 gene expression using curcumin and carnosol, well-known natural HO-1 inducers, reduces high-glucose-induced damage to retinal pigment epithelial (RPE) and human retinal endothelial (HRE) cells, respectively [7,8]. Moreover, we also demonstrated that DMF provides significant protection against high-glucose-induced damage in retinal pigment epithelial (RPE) cells through a reduction in inflammatory cascades [9]. These encouraging results from experiments carried out in in vitro models, which recapitulate oxidative stress and inflammation in the diabetic retina, suggest that DMF could reduce the inflammatory reaction and attenuate damage caused by oxidative stress associated with high levels of glucose.

Therefore, in this study, we investigated the effect of DMF on an in vivo model of diabetic retinopathy, assessing the retinal expression of the inflammatory and oxidative stress biomarkers COX-2, iNOS, and HO-1. Based on the evidence that systemically impaired glucose metabolism causes dysfunction and changes to metabolic pathways in the retina after the onset of diabetes [9], we focused our investigations on the third week after streptozotocin-induced diabetic retinopathy. Diabetic macular edema (DME), characterized by exudative fluid accumulation in the retina, is the most common form of sight-threatening retinopathy in patients with diabetes. The pharmacological treatment of DME is characterized by the intraocular administration of anti-VEGF agents such as ranibizumab or anti-inflammatory drugs such as dexamethasone. Anti-VEGF agents have a high retinal anti-permeability effect but no impact on inflammation, and this might explain the partial response in about one-third of patients [10]. On the other hand, corticosteroids, approved to handle DME, have poor safety profiles with a high risk of ocular hypertension and cataracts. The findings presented in this study suggest that DMF could play an important role in future therapeutic strategies to handle diabetic retinopathy and possibly prevent DME.

## 2. Results

### 2.1. Body Weight and Blood Glucose Levels

Starting on the first experimental day and, subsequently, once a week for the entire duration of the experimental protocol, a One-Touch Basic blood glucose Test (Accu-Check Active1, Roche Diagnostic, Milan, Italy) was used to monitor the blood glucose level in a tail-vein blood sample to confirm that STZ treatment induced hyperglycemia (Table 1). After the STZ injection and throughout the DMF treatment, animals’ body weights were monitored to avoid weight reductions of more than 10%.

### 2.2. HO-1, COX-2, and iNOS Protein Expression

**Figure 1:**
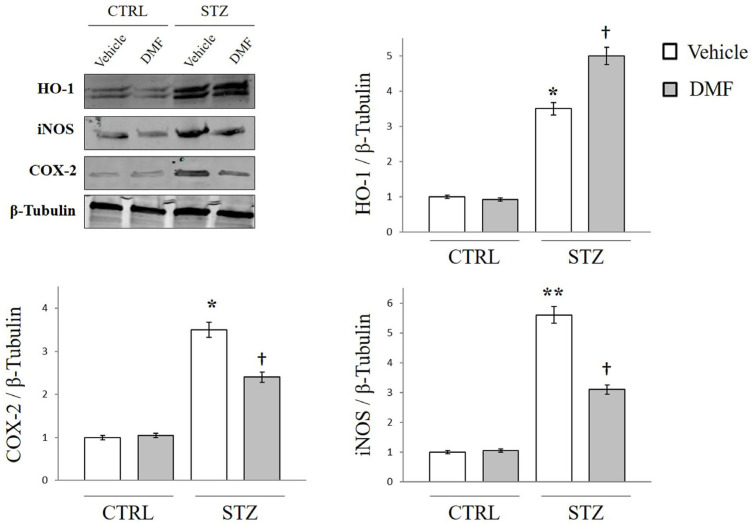
HO-1, COX-2 and iNOS protein expression in the retina of the control group (CTRL), and diabetic rats (STZ) intraperitonially injected with only Vehicle or DMF. Representative Immunoblot of signals detected by HO-1, COX-2 and iNOS antibodies obtained using 32µg of tissue homogenate from whole-retina of non-diabetic (CTRL) and diabetic (STZ) rats following intraperitoneal injection of saline (Vehicle) or DMF (DMF). The bars show the data of three independent experiments. ImageJ software was used to quantify the relative band density. Results are representative of at least three independent experiments (n = 4). Data are expressed as Mean ± SEM * *p* < 0.05 or ** *p* < 0.01 vs. CTRL, † *p* < 0.05 vs. STZ, as determined by One-Way ANOVA followed by Tukey post hoc test.

To study the effect of DMF on oxidative stress and the inflammatory process in DR, we assessed the expression profile of HO-1, COX-2, and iNOS via Western blot analysis. As shown in Figure 1, STZ-injected rats displayed a significant retinal upregulation of HO-1, COX-2, and iNOS when compared with the retina of non-diabetic rats (* *p* < 0.05 and ** *p* < 0.01 vs. CTRL). A single intraperitoneal dose of DMF in hyperglycemic rats significantly increased the retinal expression of HO-1 († *p* < 0.01 vs. STZ + Vehicle), confirming the role of DMF as an HO-1 inducer exerting antioxidant effects. Moreover, the treatment with DMF strongly reduced the expression of the inflammatory factors COX-2 and iNOS, when compared with diabetic rats († *p* < 0.05 vs. STZ + Vehicle), confirming the anti-inflammatory role played by DMF.

### 2.3. HO-1, COX-2, and iNOS Localization in Rat Retina

**Figure 2:**
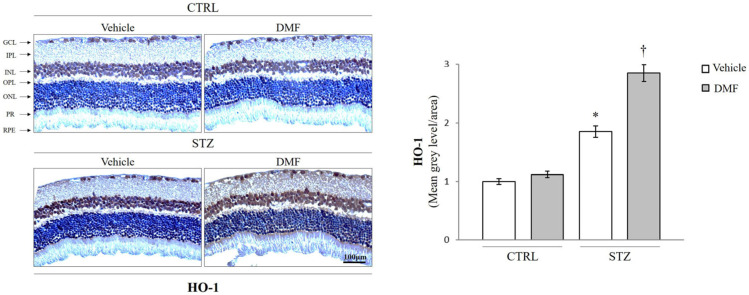
Localization of HO-1 in the retina of the control group (CTRL), and diabetic rats (STZ) intraperitonially injected with Vehicle or DMF. Representative immunohistochemical staining of HO-1 in retina of non-diabetic (CTRL) and diabetic (STZ) rats following intraperitoneal injection of saline (Vehicle) or DMF (DMF). Retinal layers are indicated as follows: ganglion cell layer (GCL), inner plexiform layer (IPL), inner nuclear layer (INL), outer plexiform (OPL), outer nuclear layers (ONL), photoreceptors layer (PR) and retinal pigmented epithelium (RPE). The bar graphs show the results of densitometric analyses. Vehicle (white bars) and DMF (gray bars). Results are representative of at least three independent experiments (n = 4). Data are expressed as Mean ± SEM * *p* < 0.05 vs. CTRL, † *p* < 0.05 vs. STZ, as determined by One-Way ANOVA followed by Tukey post hoc test.

**Figure 3:**
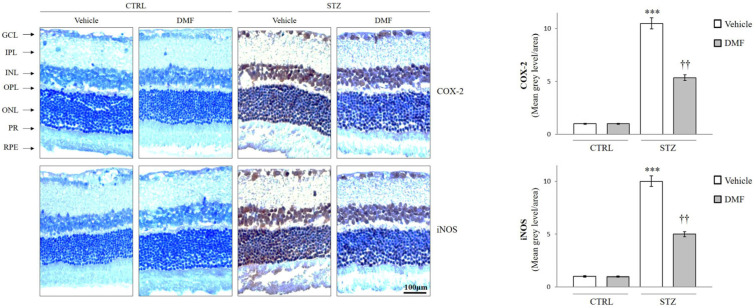
Localization of COX-2 and iNOS in the retina of the control group (CTRL), and diabetic rats (STZ) intraperitonially injected with Vehicle or DMF. Representative immunohistochemical staining of COX-2 and iNOS in the retina of control animals or diabetic rats (STZ) injected with Vehicle or after DMF treatment. Retinal layers are indicated as follows: ganglion cell layer (GCL), inner plexiform layer (IPL), inner nuclear layer (INL), outer plexiform (OPL), outer nuclear layers (ONL), photoreceptors layer (PR) and retinal pigmented epithelium (RPE). The bar graphs show the results of densitometric analyses. Vehicle (white bars) and DMF (gray bars). Results are representative of at least three independent experiments (n = 4). Data are expressed as Mean ± SEM *** *p* < 0.001 vs. CTRL, †† *p* < 0.01 vs. STZ, as determined by One-Way ANOVA followed by Tukey post hoc test.

To assess whether DMF treatment also affects the distribution of HO-1, COX-2, and iNOS in the retinal layers of hyperglycemic rats, immunohistochemistry analysis was also carried out (Figure 2 and Figure 3). As shown in Figure 2, in the control groups, HO-1 was weakly expressed in the INL and in the GCL. The HO-1 signal was more intense in the INL and the GCL of the STZ group (* *p* < 0.05 vs. CTRL), but the treatment with DMF significantly increased the expression of HO-1, not only in the INL and the GCL but also in the photoreceptor layer (PR) († *p* < 0.05 vs. STZ Vehicle, Figure 3). As shown in Figure 3, COX-2 and iNOS are weakly expressed in all retinal layers of the control group. The expression of these pro-inflammatory factors is higher in the STZ-injected rats, particularly in the GCL, INL, ONL, and the PR layer, as well as in the retinal pigmental epithelium (RPE) (*** *p* < 0.001 vs. CTRL). An intraperitoneal injection of DMF induced a significant reduction in their immunosignal in all retinal layers (†† *p* < 0.01 vs. STZ Vehicle).

## 3. Discussion

The present study demonstrates that DMF protects the rat retina against streptozotocin-induced damage via the attenuation of inflammatory cascades that involve the Nrf2/HO-1 pathway, suggesting a potential role in the treatment of diabetic retinopathy. The imbalance between oxidative stress and antioxidants is a major contributor to RPE loss of function and damage in many ocular diseases, including age-related macular degeneration (AMD) and diabetic retinopathy. RPE cells need a high amount of energy to ensure several physiological processes, such as exchanges between the choroid and retinal tissues through active transport. The primary source of energy for these cells is the β-oxidation of fatty acids, which produces many reactive oxygen species (ROS), increasing the risk of oxidative stress. Lipid peroxidation is a consequence of ROS production, and the high content of lipids in the retina puts it at high risk for oxidative damage [11]. To deal with the risk of damage mediated by ROS, RPE cells have antioxidant mechanisms, such as those mediated by the transcription factor Nrf2, which activate many target genes that regulate several processes, such as xenobiotic detoxification, redox balancing, heme metabolism, and nicotinamide adenine dinucleotide phosphate and NADPH production [12,13]. Indeed, Nrf2 has been recognized as the main regulator of protective gene transcription induced by several oxidative insults or Nrf2 activators. Under physiological conditions, Nrf2 binds Keap1—the Kelch-like ECH-associated protein 1, a cysteine-rich protein that is responsible for its cytosolic sequestration [14]. In the presence of stressors, Keap1 releases Nrf2 that can translocate into the nucleus and bind to promoters to activate gene expression [15]. The target genes include phase II detoxifying, metabolizing, and antioxidant genes (NADPH quinone oxidoreductase 1, NQO1, heme oxygenase 1, HMOX1, glutamate cysteine ligase, GLC, and glutamate S transferase, GSTs) that convert free radicals to less toxic or non-toxic elements and protect the cells against the consequences of oxidative damage. In addition, Nrf2 was found to be a negative regulator of the NADPH oxidase subunit NOX2. Nrf2^−/−^ mice showed reduced tolerance to stress in general, and ocular damage (retinal degeneration characterized by subretinal deposition of lipofuscin, loss of RPE cells, and choroidal neovascularization, and cataract) develops at 12 months of age [16]. These mice are more susceptible to retinal damage following optic nerve crush (ONC) injury and show an increase in oxidative stress markers after injury compared with the wild type [12]. Similarly, retinal ganglion cell (RGC) loss was found to be higher in Nrf2^−/−^ in the ischemia–reperfusion (I/R) model of glaucoma [17]. Moreover, the Nrf2 activator treatment was protective against RGC death in these models.

An important target of Nrf2 is the Hmox1 gene, which encodes heme oxygenase-1 (HO-1), an enzyme responsible for converting heme into biliverdin, carbon monoxide, and iron [18,19]. Heme degradation by HO-1 reduces oxidative stress, heme being a promoter of ROS production, and biliverdin and carbon monoxide antioxidants [20]. Hmox1^−/−^ mice are characterized by a very high perinatal lethality and a very severe form of anemia due to low levels of serum iron [21,22,23].

Nrf2 signaling has also been implicated in the pathogenesis of other diabetic complications, such as diabetic nephropathy: hyperglycemia increases the production of ROS, activating several downstream signaling pathways in glomerular mesangium, inducing inflammation and proliferation that cause hypertrophy of the mesangium, extracellular matrix protein accumulation and glomerular atrophy [24]. Nrf2 has been demonstrated to be crucial in maintaining the oxidant/antioxidant balance in this context, binding to the Antioxidant Response Element (ARE) at the promoter region to express genes [25,26]. Nrf2 levels are low in pre-diabetics and diabetic patients compared with non-diabetics, and this can explain the oxidative damage and diabetes complications [24].

Agents targeting the Nrf2 pathway have been investigated for their potential therapeutic effects in several diseases. Previous studies showed the beneficial effects of the induction of HO-1 in the retinal epithelium against oxidative stress derived from phototoxic damage [27] but also to reduce inflammation, promote wound healing, and protect against streptozotocin-induced damage [5,28]. The pharmacological induction of HO-1 has been demonstrated to protect retinal tissues from acute damage following the increase in ROS generation induced by the ischemia–reperfusion model [29]. Impairment in the Nrf2/HO-1 pathway has been reported in several ocular diseases, such as uveitis, cataracts, glaucoma, diabetic retinopathy, age-related macular degeneration (AMD), and RGC damage [30,31]. Recently, an interesting in vitro study [32] (on human retinal endothelial cells (HRECs) exposed to high glucose) showed that DMF activated the Nrf2 pathway, leading to an increase in HO-1 protein levels and a decrease in intracellular ROS levels. The authors showed that DMF was able to induce an increase in Nrf2 total protein levels, accompanied downstream by an upregulation of HO-1 expression, at both the mRNA and protein levels. This evidence was also supported by immunofluorescence experiments showing Nrf2 nuclear translocation. Finally, these authors found that long-term treatment with DMF, also at high concentrations (up to 50 μM), was well tolerated in HREC.

A recent study [14] supported the role of oxidative stress in a model of glaucoma and the important contribution of the endogenous antioxidant Nrf2 pathway to slow the onset of neurodegeneration after the induction of ocular hypertension and that this pathway represents an optimal therapeutic target for glaucoma therapies [12]. The knockout (KO) models for Nrf2/HO-1 exhibit retinal damage [12,16], with an increase in RGC death and levels of inflammatory markers (IL-6, TNF-α, cyclooxygenase 2, COX-2, iNOS, and monocyte chemoattractant protein 1, MCP-1) reverted by the administration of HO-1 inducers [33]. Furthermore, the overexpression of Nrf2/HO-1 in the RPE induced by the administration of an adeno-associated virus (AAV) reverted the retinal damage in mouse models of retinitis pigmentosa, resulting in slightly better visual acuity [34]. Xiong et al. demonstrated the neuroprotective effects of the delivery of Nrf2 DNA transported by an AAV that increased the endogenous antioxidant defenses, protecting photoreceptors and RGCs from oxidative stress [35]. Ildefonso et al. evaluated the effect of an Nrf2-derived peptide (a sequence of Nrf2 fused with a cell-penetrating peptide) that binds Keap-1, intravitreally delivered through an AAV [23]. This product demonstrated antioxidant and anti-inflammatory effects in different paradigms of ocular damage, an RPE oxidative injury model and a mouse model of uveitis.

DMF has been approved for the treatment of patients with psoriasis and relapsing–remitting multiple sclerosis (RRMS) due to demonstrated immuno-modulatory, anti-inflammatory, and antioxidant effects [36]. DMF binds and stabilizes Nrf2 and activates the Nrf2 pathway, thereby increasing the expression of the ARE-driven genes [37]. Therefore, DMF could be potentially repurposed as a therapeutic option for many diseases, including ocular diseases characterized by inflammation and oxidative stress [4]. In vivo studies demonstrated an increase in the level of Nrf2 and NQO1 activity in the central nervous system of animals treated with DMF. Previous studies showed the neuroprotective role of the active metabolite of DMF, monomethyl fumarate (MMF), in wild-type C57BL/6J and Nrf2 knockout (KO) mice with retinal ischemia–reperfusion (I/R) injury [38]. MMF significantly induced antioxidative genes and reduced inflammatory gene expression, decreased cell loss, and improved retinal function after retinal damage in wild-type mice but not in Nrf2 KO mice, suggesting that Nrf2 modulation plays a major role in retinal protection. Recently, Mori et al. [39] showed that DMF ameliorates RGC survival after optic nerve crush, possibly through the Nrf2/HO-1 axis. DMF treatment prevented retinal injury in a model of light-induced photoreceptor loss, which resulted in retinal degeneration tightly linked to oxidative stress and made the damage sensitive to the antioxidant mode of action of the drug [37]. Moreover, the drug has been tested in patients to study the effects of oral administration of DMF (120 mg DMF twice daily for the first week, and then 240 mg DMF twice daily for 51 weeks) in patients with central or non-central geographic atrophy (GA), compared with a cohort of subjects with AMD with no specific treatment [4]. Novel delivery systems of DMF for eye conditions are expected, with the aim of improving ocular bioavailability.

In line with this evidence, the present study demonstrated a significant increase in HO-1 levels in all retinal layers of the STZ group. Moreover, in our experimental model, the rise in HO-1 was greater in the INL and the GCL. This increase was further raised by DMF treatment, with the largest effect observed in the retinal INL, GCL, and PR layers. This greater activation of HO-1 induced a parallel strong reduction in the inflammatory protein expression (COX-2 and iNOS) in all retinal layers of STZ-injected rats treated with DMF in comparison with those treated only with vehicle.

The KO models for Nrf2/HO-1 exhibited retinal damage [12,16], with an increase in RGC death and increased levels of inflammatory markers (IL-6, TNF-α, cyclooxygenase 2, COX-2, iNOS, and monocyte chemoattractant protein 1, MCP-1), reverted by the administration of HO-1 inducers [33]. Mori et al. [39] showed that DMF promotes the survival of RGCs after optic nerve crush, possibly via the Nrf2/HO-1 pathway. Moreover, the drug is under clinical investigation (www.clinicaltrial.gov NCT04292080) to assess the effects of oral administration of DMF (120 mg DMF twice a day for the first week, and then 240 mg DMF twice a day for 51 weeks) in patients with central or non-central retinal GA in comparison with a cohort of subjects with AMD with no specific treatment.

In this study, we observed a remarkable increase in HO-1 levels in all retinal layers of the STZ group, especially in the INL and GCL, as a physiological protective mechanism of retinal tissue. This increase was potentiated by DMF treatment, with the largest effect observed in the retinal INL, GCL, and PR layers. This greater activation of HO-1 induced a remarkable parallel reduction in the inflammatory protein expression (COX-2 and iNOS) in all retinal layers of STZ-injected rats treated with DMF in comparison with those treated only with vehicle.

## 4. Materials and Methods

### 4.1. Animals

Male Sprague-Dawley adult rats (weighing 200–250 g) were obtained from Charles River (Calco, Italy) and were handled according to the ARVO Statement for the Use of Animals in Ophthalmic and Vision Research and the Directive 2010/63/EU of the European Parliament and of the Council. The protocol was approved by the Institutional Animal Care and Use Committee of the University of Catania (#279). The animals were fed on standard laboratory food and were allowed free access to water in a light- and temperature-controlled room with a 12 h light/12 h dark cycle. Animals were randomly assigned to the following experimental groups (n = 8 each group): (1) Control (Non-diabetic) + Vehicle; (2) Control (Non-diabetic) + DMF i.p. (10 mg/Kg); (3) Diabetic + Vehicle; (4) Diabetic + DMF i.p. (10 mg/Kg).

### 4.2. Induction of Diabetes

Induction of diabetes was obtained by a single intraperitoneal (i.p.) injection (of streptozotocin (STZ, 60 mg/kg, purchased from Sigma–Aldrich, Milan, Italy). The diabetic state was confirmed by daily measurement of glycemia (with a blood glucose meter, Accu-Check Active1, Roche Diagnostic, Milan, Italy), and diabetic rats (blood glucose levels > 250 mg/dL) were randomly assigned to the vehicle or treatment groups. Body weight was monitored during the study (no more than 10% body weight loss was observed in diabetic rats). Non-diabetic control animals received injections (i.p.) of vehicle (citrate buffer alone).

### 4.3. DMF Treatment

Dimethyl fumarate (DMF) was administered via a daily i.p. injection at a dosage of 10 mg/kg for 3 weeks. At the end of the treatment, the rats were killed by CO_2_ inhalation, some eyes were enucleated and fixed with 4% paraformaldehyde for immunohistochemical staining, and the rest of the eyes were used to collect retina samples, stored in RIPA buffer, for Western blot analysis.

### 4.4. Western Blot Analysis

Western blot analysis was carried out to assess the relative expression levels of HO-1, COX-2, and iNOS in rat retinal tissue, as previously described [40,41]. Briefly, protein extraction was performed by using RIPA buffer (Thermo Fisher Scientific, Paisley, UK) in the presence of phosphatase and protease inhibitors (Thermo Fisher Scientific, Paisley, UK). Subsequently, each sample was homogenized and sonicated twice for 20 s using an ultrasonic probe. Quant-iT Protein Assay Kit (Thermo Fisher Invitrogen, Waltham, MA, USA) was used to determine the protein concentration of the samples, and 32 μg of each was processed and separated on a Biorad Criterion XT 4–15% Bis-tris gel (BIO-RAD) via electrophoresis and transferred to a nitrocellulose membrane (BIO-RAD, Hercules, CA, USA). Blots were blocked using Odyssey Blocking Buffer (LI-COR Biosciences, Lincoln, NE, USA) and probed with appropriate antibodies: COX-2 (sc-19999, Santa Cruz Biotechnology, Inc., Santa Cruz, CA, USA), iNOS (sc-651, Santa Cruz Biotechnology, Inc., Santa Cruz, CA, USA), and HO-1 (#GXGTX101147, GeneTex, Irvine, CA, USA). The secondary antibody goat anti-rabbit IRDye 800CW (#926-32211; LI-COR Biosciences, Lincoln, NE, USA) and goat anti-mouse IRDye 680CW, (#926-68020D; LI-COR Biosciences, Lincoln, NE, USA) were used at 1:20,000. Blots were scanned with an Odyssey Infrared Imaging System (Odyssey). Densitometry analyses of blots were performed at non-saturating exposures and analyzed using ImageJ software (NIH, Bethesda, MD, USA; available at http://rsb.info.nih.gov/ij/index.html). Values were normalized to β-tubulin, which was used as the loading control.

### 4.5. Tissue Preparation for Retinal Histology and Immunohistochemical Staining (IHC)

Eyes were enucleated and fixed overnight with 4% paraformaldehyde in 0.1 M sodium phosphate (pH 7.6). The paraffin-embedded retina sections were stained with hematoxylin–eosin, and the micrographs were photographed at 40× magnification under a light microscope (Axiovert, Carl Zeiss Inc., Cambridge, UK). IHC analyses were performed on whole retina, as previously described [42]. Briefly, retinal sections were dewaxed in xylene, hydrated using graded ethanol, incubated in 0.3% H_2_O_2_/methanol solution for 30 min, and then rinsed with phosphate-buffered saline (PBS; Sigma, Milan, Italy). To reduce non-specific staining, sections were treated with 1% bovine serum albumin (BSA) in PBS for 1 h and incubated with specific antibodies (for catalog number see “Western blot analysis” section). After 24 h, HRP-conjugated were used as secondary antibodies, the immunoreaction was observed using 3,3′-diaminobenzidine solution (DAB), and then hematoxylin was used as the nuclear counterstain. The sections were examined with a Zeiss Axioplan light microscope (Carl Zeiss, Oberkochen, Germany) and photographed with a digital camera (AxioCam MRc5, Carl Zeiss, Oberkochen, Germany). Densitometric analysis of the immunohistochemical staining was performed with ImageJ software. HO-1, COX-2, and iNOS staining in rat retinas was quantified as follows: (i) images were converted to black and white; (ii) blue channel color was switched off; (iii) average gray values were then normalized to the control group.

### 4.6. Statistical Analysis

Data are reported as mean ± S.E.M. One-way analysis of variance (ANOVA) was used to compare the differences among groups, and statistical significance was assessed using the Tukey post hoc test. The level of significance for all statistical tests was *p* ≤ 0.05.

## 5. Conclusions

The present data demonstrate that DMF treatment protects rat retina against streptozotocin-induced damage via counteracting oxidative stress and inflammation. In conclusion, we believe that DMF represents a potential candidate to treat diabetic retinopathy and warrants further in vivo and clinical evaluation.

## Figures and Tables

**Table 1:** Effects of STZ-induced diabetes on body weight and blood glucose levels after 1 and 3 weeks. CTRL (non-diabetic) + vehicle, CTRL (non-diabetic) + DMF, STZ injected (diabetic) + vehicle, STZ injected (diabetic) + DMF injected. Data are expressed as mean ± SD. * *p* < 0.05 vs. CTRL; ** *p* < 0.01 vs. CTRL.

Groups	Body Weight 1 Week (g)	Body Weight 3 Weeks (g)	Non-Fasting Glucose 1 Week (mg/dL)	Non-Fasting Glucose 3 Weeks (mg/dL)
*CTRL + vehicle*	245 ± 15	250 ± 12	106 ± 12	104 ± 14
*CTRL + DMF*	242 ± 13	253 ± 11	105 ± 10	107 ± 9
*STZ + vehicle*	235 ± 12	229 ± 14 *	321 ± 15 **	312 ± 28 **
*STZ + DMF*	239 ± 13	231 ± 12 *	332 ± 15 **	343 ± 10 **

## Data Availability

Data is contained within the article.

## References

[1] Gomułka K., Ruta M. (2023). The Role of Inflammation and Therapeutic Concepts in Diabetic Retinopathy—A Short Review. Int. J. Mol. Sci..

[2] Gagliano C., Caruso S., Napolitano G., Malaguarnera G., Cicinelli M.V., Amato R., Reibaldi M., Incarbone G., Bucolo C., Drago F. (2014). Low levels of 17-β-oestradiol, oestrone and testosterone correlate with severe evaporative dysfunctional tear syndrome in postmenopausal women: A case–control study. Br. J. Ophthalmol..

[3] Mallucci G., Annovazzi P., Miante S., Torri-Clerici V., Matta M., La Gioia S., Cavarretta R., Mantero V., Costantini G., D’ambrosio V. (2018). Two-year real-life efficacy, tolerability and safety of dimethyl fumarate in an Italian multicentre study. J. Neurol..

[4] Manai F., Govoni S., Amadio M. (2022). The Challenge of Dimethyl Fumarate Repurposing in Eye Pathologies. Cells.

[5] Fan J., Xu G., Jiang T., Qin Y. (2012). Pharmacologic Induction of Heme Oxygenase-1 Plays a Protective Role in Diabetic Retinopathy in Rats. Investig. Opthalmology Vis. Sci..

[6] Su Q., Dong J., Zhang D., Yang L., Roy R. (2022). Protective Effects of the Bilobalide on Retinal Oxidative Stress and Inflammation in Streptozotocin-Induced Diabetic Rats. Appl. Biochem. Biotechnol..

[7] Shi Q., Wang J., Cheng Y., Dong X., Zhang M., Pei C. (2020). Palbinone alleviates diabetic retinopathy in STZ-induced rats by inhibiting NLRP3 inflammatory activity. J. Biochem. Mol. Toxicol..

[8] D’agata V., D’amico A.G., Maugeri G., Bucolo C., Rossi S., Giunta S. (2022). Carnosol attenuates high glucose damage in human retinal endothelial cells through regulation of ERK/Nrf2/HO-1 pathway. J. Asian Nat. Prod. Res..

[9] Maugeri G., Bucolo C., Drago F., Rossi S., Di Rosa M., Imbesi R., D’agata V., Giunta S. (2021). Attenuation of High Glucose-Induced Damage in RPE Cells through p38 MAPK Signaling Pathway Inhibition. Front. Pharmacol..

[10] Figueira J., Henriques J., Carneiro Â., Marques-Neves C., Flores R., Castro-Sousa J.P., Meireles A., Gomes N., Nascimento J., Amaro M. (2021). Guidelines for the Management of Center-Involving Diabetic Macular Edema: Treatment Options and Patient Monitorization. Clin. Ophthalmol..

[11] Domènech E.B., Marfany G. (2020). The Relevance of Oxidative Stress in the Pathogenesis and Therapy of Retinal Dystrophies. Antioxidants.

[12] Naguib S., Backstrom J.R., Gil M., Calkins D.J., Rex T.S. (2021). Retinal oxidative stress activates the NRF2/ARE pathway: An early endogenous protective response to ocular hypertension. Redox Biol..

[13] He M., Pan H., Chang R.C.-C., So K.-F., Brecha N.C., Pu M. (2014). Activation of the Nrf2/HO-1 Antioxidant Pathway Contributes to the Protective Effects of Lycium Barbarum Polysaccharides in the Rodent Retina after Ischemia-Reperfusion-Induced Damage. PLoS ONE.

[14] Kang M.-I., Kobayashi A., Wakabayashi N., Kim S.-G., Yamamoto M. (2004). Scaffolding of Keap1 to the actin cytoskeleton controls the function of Nrf2 as key regulator of cytoprotective phase 2 genes. Proc. Natl. Acad. Sci. USA.

[15] Baird L., Dinkova-Kostova A.T. (2011). The cytoprotective role of the Keap1–Nrf2 pathway. Arch. Toxicol..

[16] Zhao Z., Chen Y., Wang J., Sternberg P., Freeman M.L., Grossniklaus H.E., Cai J. (2011). Age-Related Retinopathy in NRF2-Deficient Mice. PLoS ONE.

[17] Xu Z., Cho H., Hartsock M.J., Mitchell K.L., Gong J., Wu L., Wei Y., Wang S., Thimmulappa R.K., Sporn M.B. (2015). Neuroprotective role of Nrf2 for retinal ganglion cells in ischemia-reperfusion. J. Neurochem..

[18] Loboda A., Damulewicz M., Pyza E., Jozkowicz A., Dulak J. (2016). Role of Nrf_2_/HO_-1_ system in development, oxidative stress response and diseases: An evolutionarily conserved mechanism. Cell. Mol. Life Sci..

[19] Reichard J.F., Motz G.T., Puga A. (2007). Heme oxygenase-1 induction by NRF2 requires inactivation of the transcriptional repressor BACH1. Nucleic Acids Res..

[20] Consoli V., Sorrenti V., Grosso S., Vanella L. (2021). Heme Oxygenase-1 Signaling and Redox Homeostasis in Physiopathological Conditions. Biomolecules.

[21] Šmíd V., Šuk J., Kachamakova-Trojanowska N., Jašprová J., Valášková P., Józkowicz A., Dulak J., Šmíd F., Vítek L., Muchová L. (2018). Heme Oxygenase-1 May Affect Cell Signalling via Modulation of Ganglioside Composition. Oxidative Med. Cell. Longev..

[22] Poss K.D., Tonegawa S. (1997). Heme oxygenase 1 is required for mammalian iron reutilization. Proc. Natl. Acad. Sci. USA.

[23] Ildefonso C.J., Jaime H., Brown E.E., Iwata R.L., Ahmed C.M., Massengill M.T., Biswal M.R., Boye S.E., Hauswirth W.W., Ash J.D. (2016). Targeting the Nrf2 Signaling Pathway in the Retina with a Gene-Delivered Secretable and Cell-Penetrating Peptide. Investig. Opthalmology Vis. Sci..

[24] Adelusi T.I., Du L., Hao M., Zhou X., Xuan Q., Apu C., Sun Y., Lu Q., Yin X. (2020). Keap1/Nrf2/ARE signaling unfolds therapeutic targets for redox imbalanced-mediated diseases and diabetic nephropathy. Biomed. Pharmacother..

[25] Kessler R.C., Adler L.A., Gruber M.J., Sarawate C.A., Spencer T., Van Brunt D.L. (2007). Validity of the World Health Organization Adult ADHD Self-Report Scale (ASRS) Screener in a representative sample of health plan members. Int. J. Methods Psychiatr. Res..

[26] Tu W., Wang H., Li S., Liu Q., Sha H. (2019). The Anti-Inflammatory and Anti-Oxidant Mechanisms of the Keap1/Nrf2/ARE Signaling Pathway in Chronic Diseases. Aging Dis..

[27] Gao X., Talalay P. (2004). Induction of phase 2 genes by sulforaphane protects retinal pigment epithelial cells against photooxidative damage. Proc. Natl. Acad. Sci. USA.

[28] Patil K., Bellner L., Cullaro G., Gotlinger K.H., Dunn M.W., Schwartzman M.L. (2008). Heme Oxygenase-1 Induction Attenuates Corneal Inflammation and Accelerates Wound Healing after Epithelial Injury. Investig. Opthalmology Vis. Sci..

[29] Sun M.-H., Pang J.-H.S., Chen S.-L., Han W.-H., Ho T.-C., Chen K.-J., Kao L.-Y., Lin K.-K., Tsao Y.-P. (2010). Retinal Protection from Acute Glaucoma-Induced Ischemia-Reperfusion Injury through Pharmacologic Induction of Heme Oxygenase-1. Investig. Opthalmology Vis. Sci..

[30] Nagai N., Thimmulappa R.K., Cano M., Fujihara M., Izumi-Nagai K., Kong X., Sporn M.B., Kensler T.W., Biswal S., Handa J.T. (2009). Nrf2 is a critical modulator of the innate immune response in a model of uveitis. Free. Radic. Biol. Med..

[31] Zyla K., Larabee C.M., Georgescu C., Berkley C., Reyna T., Plafker S.M. (2019). Dimethyl fumarate mitigates optic neuritis. Mol. Vis..

[32] Manai F., Amadio M. (2022). Dimethyl Fumarate Triggers the Antioxidant Defense System in Human Retinal Endothelial Cells through Nrf2 Activation. Antioxidants.

[33] Qi X., Walton D.A., Plafker K.S., Boulton M.E., Plafker S.M. (2022). Sulforaphane recovers cone function in an Nrf2-dependent manner in middle-aged mice undergoing RPE oxidative stress. Mol. Vis..

[34] Wu D.M., Ji X., Ivanchenko M.V., Chung M., Piper M., Rana P., Wang S.K., Xue Y., West E., Zhao S.R. (2021). Nrf2 overexpression rescues the RPE in mouse models of retinitis pigmentosa. J. Clin. Investig..

[35] Xiong W., Garfinkel A.E.M., Li Y., Benowitz L.I., Cepko C.L. (2015). NRF2 promotes neuronal survival in neurodegeneration and acute nerve damage. J. Clin. Investig..

[36] Gold R., Kappos L., Arnold D.L., Bar-Or A., Giovannoni G., Selmaj K., Tornatore C., Sweetser M.T., Yang M., Sheikh S.I. (2012). Placebo-Controlled Phase 3 Study of Oral BG-12 for Relapsing Multiple Sclerosis. N. Engl. J. Med..

[37] Dietrich M., Hecker C., Nasiri M., Samsam S., Issberner A., Kohne Z., Hartung H.-P., Albrecht P. (2020). Neuroprotective Properties of Dimethyl Fumarate Measured by Optical Coherence Tomography in Non-inflammatory Animal Models. Front. Neurol..

[38] Cho H., Hartsock M.J., Xu Z., He M., Duh E.J. (2015). Monomethyl fumarate promotes Nrf2-dependent neuroprotection in retinal ischemia-reperfusion. J. Neuroinflammation.

[39] Mori S., Kurimoto T., Maeda H., Nakamura M. (2020). Dimethyl Fumarate Promotes the Survival of Retinal Ganglion Cells after Optic Nerve Injury, Possibly through the Nrf2/HO-1 Pathway. Int. J. Mol. Sci..

[40] D’Amico A.G., Maugeri G., Magrì B., Giunta S., Saccone S., Federico C., Pricoco E., Broggi G., Caltabiano R., Musumeci G. (2023). Modulatory activity of ADNP on the hypoxia-induced angiogenic process in glioblastoma. Int. J. Oncol..

[41] Lazzara F., Fidilio A., Platania C.B.M., Giurdanella G., Salomone S., Leggio G.M., Tarallo V., Cicatiello V., De Falco S., Eandi C.M. (2019). Aflibercept regulates retinal inflammation elicited by high glucose via PlGF/ERK pathway. Biochem. Pharmacol..

[42] Romano G.L., Platania C.B.M., Leggio G.M., Torrisi S.A., Giunta S., Salomone S., Purrello M., Ragusa M., Barbagallo C., Giblin F.J. (2020). Retinal biomarkers and pharmacological targets for Hermansky-Pudlak syndrome 7. Sci. Rep..

